# Lateral connections synchronize population activity in a spiking neural network model of midbrain superior colliculus

**DOI:** 10.1186/1471-2202-16-S1-P273

**Published:** 2015-12-18

**Authors:** Bahadir Kasap, John van Opstal

**Affiliations:** 1 Donders Institute for Brain, Cognition and Behaviour; Dept. Biophysics, Radboud University Nijmegen, Nijmegen, the Netherlands

## 

Saccades are rapid and ballistic eye-head gaze shifts between points of interest in the visual field. They are crucial for gathering high-resolution visual information. The midbrain superior colliculus (SC) generates saccadic eye-movement commands for downstream oculomotor circuits. It contains an eye-centered, gaze-motor map that relates the location of a Gaussian-shaped neural population to the intended movement vector. The gaze-motor map mediates the spatiotemporal transformation for eye-head orienting gaze shifts to peripheral targets [1]. Electrophysiological recordings have shown that SC neurons exhibit some remarkable activity properties that depend on both their anatomical position and the resulting saccade trajectory [2].

Here, we propose a biologically plausible spiking neural network model that is constrained by the observed firing patterns of real SC neurons for visually evoked saccades. The functional two-dimensional network model reproduces the spike trains of single neurons in recorded SC populations for saccades with different amplitudes and directions.

The network model consists of a 2D grid of neurons, representing the gaze-motor map. The adaptive integrate-and-fire neurons [3] portray the observed site-dependent bursting profiles of individual SC neurons through distinct intrinsic biophysical properties, whereas Mexican-hat shaped lateral connections ensure the observed synchronized population activity by a soft winner-takes-all mechanism.

We argue that our model offers a basis for neuronal algorithms of spatiotemporal transformations and bio-inspired optimal control signal generators.
